# Mr. David Rice

**Published:** 1910-06-04

**Authors:** 


We have to record the death of Mr. David Rice, at the age of seventy-three. Mr. Rice, who studied at Leeds, took his diploma in 1858, and became an L.S.A. in 1861. In after years he made his home at Southam in Warwickshire.

